# Network pharmacology-based prediction of active compounds in the Wenyang Jiedu Huayu formula acting on acute-on-chronic liver failure with experimental support *in vitro* and *in vivo*

**DOI:** 10.3389/fphar.2022.1003479

**Published:** 2022-10-20

**Authors:** Dan Tang, Ruo-Yu Wang, Ke-Wei Sun, Yunan Wu, Lin Ding, Yang Mo

**Affiliations:** ^1^ Department of Hepatology, The First Hospital of Hunan University of Traditional Chinese Medicine, Changsha, China; ^2^ Academic Affairs Office, Hunan University of Traditional Chinese Medicine, Changsha, China

**Keywords:** acute-on-chronic liver failure, Wenyang Jiedu Huayu formula, network pharmacology, atractylenolide I, rat hepatocytes, ACLF rat model

## Abstract

Acute-on-chronic liver failure (ACLF) is characterized by undermined liver function, massive necrosis/apoptosis of hepatocytes, and hepatic inflammatory cell recruitment, leading to multiorgan failure. Traditional Chinese medicine (TCM) has been widely applied in clinical and experimental studies of ACLF. In this study, 23 compounds with 6,386 drug targets were obtained from Wenyang Jiedu Huayu (WYJDHY), and 8,096 genes were identified as ACLF disease targets, among which 3,132 were overlapping co-targets. Expression profile analysis identified 105 DEGs among the co-targets, which were associated with biological activities such as lymphocyte activation, immune response regulation, and pathways such as Th17 cell differentiation and NF-κB signaling. After PPI analysis and network construction, atractylenolide I (AT-1) has been identified as the hub active ingredient of the WYJDHY formula. LPS stimulation inhibited rat hepatocytes’ BRL 3A cell viability, promoted cell apoptosis, increased the levels of ALT, AST, IL-6, and VCAM-1 within the culture medium, and activated NF-κB signaling, whereas AT-1 treatment significantly attenuated LPS-induced toxicity on BRL 3A cells. Furthermore, the NF-κB signaling inhibitor PDTC exerted effects on LPS-stimulated BRL 3A cells similar to those of AT-1, and the combination of PDTC and AT-1 further attenuated LPS-induced toxicity on BRL 3A cells. *In vivo*, AT-1 alone or with PDTC improved the symptoms and local inflammation in ACLF model rats. In conclusion, 23 active ingredients of six herbs in the WYJDHY formula were retrieved, and 105 co-targets were differentially expressed in ACLF. AT-1 exerts protective effects on LPS-stimulated hepatocytes and ACLF rats, possibly by inhibiting the NF-κB pathway.

## Introduction

Acute-on-chronic liver failure (ACLF) is characterized by undermined liver function and massive necrosis/apoptosis of hepatocytes and hepatic inflammatory cell recruitment, resulting in multiorgan failure (Arroyo et al., 2015). Chronic liver disease, severe decompensation, and liver failure occurring in a short amount of time might result in ACLF (Liver et al., 2019). Hepatitis B virus (HBV)-induced liver failure is the most frequent form of liver diseases, with ACLF being the most common clinical manifestation, accounting for 80–90% of cases in China (Liver et al., 2019), with a high mortality rate, numerous complications, and extremely limited treatment (Liver et al., 2013; Kabbani et al., 2021). Despite significant interventions, the short-term rates of liver failure mortality remain unacceptable, owing mostly to insufficient response to standard therapy and a dearth of liver transplantation options (Bernal et al., 2015). Moreover, some of the illness management practices following conventional Western medicine treatment are inadequate, leaving clinical needs unmet (Gao et al., 2017).

Numerous factors, such as endotoxin and pro-inflammatory cytokines, have been linked to the disease’s pathogenesis (Zhou M. et al., 2019; Kessoku et al., 2021; Lad et al., 2021). Host immunity, resulting in the release of pro-inflammatory cytokines and activation of the excessive immune cascade, is accompanied by significant liver damage in the early stages of ACLF (Liu, 2009). Pro-inflammatory cytokines, such as tumor necrosis factor (TNF), gradually increase in correlation with ACLF’s severity and prognosis (Zou et al., 2009; Wang Z. L. et al., 2016). Toll-like receptor (TLR) pathways might also influence the activation of multiple immediate early genes associated with the pro-inflammatory cytokines in the process of liver failure (Sun et al., 2009; McCabe et al., 2012). Moreover, as a critical factor of the TLR pathway, NF-κB signaling exerts crucial effects on TNF cascades (Napetschnig and Wu, 2013). The Akt pathway is tightly associated with immune modulation after TLR activation (Zhang and Lu, 2015). Several reagents have been reported to alleviate ACLF by regulating the NF-κB pathway, including trichostatin A (Zhang et al., 2015), ginsenoside Rg1 (Zhao et al., 2021), and plumbagin (Wang H. et al., 2016). Furthermore, the NF-κB pathway has been implicated in the progression of ACLF. Zhang et al. (2022b) demonstrated that NF-κB activation and increased inflammation participated in the pathogenesis of HBV–ACLF injury. Yang et al. (2014) reported that the inhibition of NF-κB and TNF-α resulted in different effects on ACLF rats. Therefore, identifying more efficient reagents modulating the NF-κB pathway might provide promising candidates for ACLF treatment.

In recent years, TCM has been widely used in clinical and experimental studies of ACLF; it has been confirmed to have some advantages in protecting liver function and delaying disease progression (Chen et al., 2019). The Wenyang Jiedu Huayu (WYJDHY) formula, which is composed of FuZi (Aconiti Lateralis Radix Praeparata), BaiZhu (Atractylodis Macrocephalae Rhizoma), Chishao (Radix Paeoniae Rubra), YinChen (Artemisiae Scopariae Herba), DanShen (*Salvia miltiorrhiza* Bunge), and YiYiRen (*Coix lacryma-jobi L*.), has long been used for jaundice and liver failure treatment in TCM. The WYJDHY formula could improve liver failure patients’ liver function and blood coagulation function (Zuo, 2021). In this formula, Chishao is bitter and slightly cold in nature, which is used to clear heat and cool the blood to resolve blood stasis, whereas FuZi warms the spleen and kidneys to prevent the bitter cold from injuring the stomach, and these two are the monarch drugs in the formula. The combination of YinChen and DanShen is the subject herb to strengthen the effect of clearing heat and dampness and activating blood circulation to remove blood stasis. Then, BaiZhu and YiYiRen are used to strengthen the spleen and dry dampness to prevent the drugs from damaging the spleen and stomach. The whole formula is based on the treatment method of “clearing heat and dampness, cooling blood, detoxifying, and activating blood stasis” for jaundice in liver failure, emphasizing warming Yang and strengthening the spleen. The experimental studies confirmed (Chen et al., 2013; Wang et al., 2013) that WYJDHY, detoxifying and resolving blood stasis, can increase intestinal sIgA secretion, lower serum endotoxin levels, and reduce hepatic NF-κB expression. Our previous study has also confirmed that the warming Yang method using BaiZhu and FuZi could enhance the therapeutic effect of traditional Chinese medicine decoction on ACLF (Sun et al., 2010). However, the key active ingredients of these herbs’ protection, especially BaiZhu and FuZi, against ACLF and the underlying mechanism remain unclear.

Network pharmacology is a novel multi-target therapeutic molecule design technique based on systems biology theory, network analysis of biological systems, and identifying particular signal nodes (Khanal and Patil, 2021; Zeng et al., 2022). It focuses on multimodal regulation of signal transduction pathways to maximize therapeutic efficacy while avoiding collateral side responses (Hopkins, 2008). Notably, TCM and global ethnic medicine target different molecules in the human body to achieve curative effectiveness (Hao da and Xiao, 2014; Zhou et al., 2020; Jiao et al., 2021). As a result, network-based pharmacology techniques in TCM research may be viable options for investigating the mechanism of WYJDHY treating ACLF. We searched TCMSP to acquire the major active ingredients of herbs in WYJDHY, identified drug and disease targets, used GEO expression profiles to analyze differentially expressed genes (DEGs) between ACLF and healthy samples, and identified overlapping genes as hub genes. The herb–active ingredient–signaling pathway–target networks were constructed. Atractylenolide I (AT-1) and its targeted pathway NF-κB were chosen for cellular experiments. The effects of AT-1 on LPS-stimulated rat hepatocytes were investigated, and the involvement of NF-κB signaling was evaluated.

## Materials and methods

### Retrieval of compound information in WYJDHY

The Traditional Chinese Medicine System Pharmacology Database (TCMSP, http://lsp.nwu.edu.cn/tcmsp.php) was used to collect the chemical ingredients of BaiZhu, Chishao, DanShan, YiYiRen, YinChen, and FuZi (Table 1) (Ru et al., 2014). The active compounds were screened based on absorption, distribution, metabolism, and excretion (ADME), and bio-active components were recovered for further study using pharmacokinetic information retrieval filters under screening conditions of OB 20% (Xu et al., 2012) and DL ≥ 0.1 (Jia et al., 2020).

**TABLE 1:** Main active ingredients with targeted genes in herbs of the Wenyang Jiedu Huayu formula, according to the TCMSP database and PubChem database.

TCM	Mol ID	Molecule name	MW	OB(%)	DL
BaiZhu	MOL000038	Acridine	179.23	33.71	0.1
	MOL000043	Atractylenolide I	230.33	37.37	0.15
	MOL000057	Diisobutyl phthalate	278.38	49.63	0.13
Chishao	MOL002714	Baicalein	270.25	33.52	0.21
	MOL002776	Baicalin	446.39	40.12	0.75
	MOL000358	Beta-sitosterol	414.79	36.91	0.75
	MOL000492	Cianidanol	290.29	54.83	0.24
	MOL000449	Stigmasterol	412.77	43.83	0.76
Danshen	MOL002776	Baicalin	446.39	40.12	0.75
	MOL007088	Cryptotanshinone	296.39	52.34	0.4
	MOL000569	Digallic acid	322.24	61.85	0.26
	MOL001942	Isoimperatorin	270.3	45.46	0.23
	MOL000006	Luteolin	286.25	36.16	0.25
	MOL007122	Miltirone	282.41	38.76	0.25
	MOL007154	Tanshinone IIA	294.37	49.89	0.4
YiYiRen	MOL000953	Cholesterol	386.73	37.87	0.68
	MOL001884	Demecolcine	371.47	26.6	0.51
	MOL000508	Friedelin	426.8	29.16	0.76
	MOL000449	Stigmasterol	412.77	43.83	0.76
Yinchen	MOL000358	Beta-sitosterol	414.79	36.91	0.75
	MOL007274	Cirsimaritin	314.31	30.35	0.3
	MOL005573	Genkwanin	284.28	37.13	0.24
	MOL000354	Isorhamnetin	316.28	49.6	0.31
	MOL000098	Quercetin	302.25	46.43	0.28
FuZi	MOL002398	Karanjin	292.3	69.56	0.34
	MOL000131	Linoleic acid	280.5	41.9	0.14

### Retrieval of compound targets and disease targets

PubChem (https://pubchem.ncbi.nlm.nih.gov/) and PubMed (https://pubmed.ncbi.nlm.nih.gov/) were used to find the protein targets of the active chemicals in BaiZhu, Chishao, DanShan, YiYiRen, YinChen, and FuZi.

Searching for ‘ACLF’ on GeneCards and comparing differentially expressed genes (DEGs) between ACLF and normal samples yielded the disease target genes of ACLF.

### DEGs between ACLF and normal samples

GSE142255 was downloaded and analyzed for DEGs between seven cases of normal control and nine cases of ACLF samples combined by the metaMA package. The threshold was set as |logFC|>1 and FDR<0.05.

### Gene Ontology functional and KEGG signaling pathway enrichment annotation for 105 hub genes

The GO and KEGG signaling enrichment analyses were conducted using the functional annotation tool of Metascape (https://metascape.org/gp/) (Zhou Y. et al., 2019). Terms with thresholds of Count ≥2 and Expression Analysis Systematic Explorer (EASE) scores ≤0.05 were chosen in functional annotation clustering.

### Protein–protein interaction analysis

PPI data were retrieved from STRING (https://string-db.org/cgi/input.pl), and a physical score >0.132 was applied to construct a PPI network. Only interactions with weight greater than the threshold were chosen for the newly built PPI network.

### Construction and analysis of networks

The ingredient–target–pathway networks were constructed by Cytoscape_v3.8.0 (Smoot et al., 2011). In these graphical networks, the herbs, compounds, pathways, or targets were represented by nodes, while the compound–target or target–pathway interactions were represented by edges.

### Cell lineage and LPS stimulation

The rat hepatocyte cell line BRL 3A was procured from Procell (CL-0038, China) and cultivated in Dulbecco’s modified Eagle’s medium (DMEM, Gibco, Carlsbad, CA, United States) added with 10% FBS (Gibco). For the cellular inflammation model induction, BRL 3A cells were treated with 1 μg/ml LPS for 24 h.

### The maximum non-cytotoxic concentration of atractylenolide I (AT-1)

BRL 3A cells were treated for 24 and 48 h with gradient doses of AT-1 (0, 1, 5, 10, 20, 40, and 80 μM, obtained from Xiya Reagent, China). CCK-8 assays were subsequently conducted to examine cell viability, and the maximum non-cytotoxic concentration of AT-1 was selected.

### Cell counting kit-8

BRL 3A cells were seeded onto 96-well plates and cultured with AT-1 for 24 h (with or without LPS) for cell viability determination. The vitality of the cells was then determined using CCK-8, as directed by the manufacturer. In each well, 10 μl of cell counting kit solution was supplemented, followed by the incubation of 96-well plates at 37°C for 2 h. A microplate reader at 490 nm was then used to measure the absorbencies.

### Flow cytometry

An annexin-V-fluorescein isothiocyanate (FITC) kit (BD Biosciences, San Jose, CA, United States) was used to measure the proportion of cells undergoing apoptosis. BRL 3A cells were treated with AT-1, collected, resuspended in 500 μl augmented binding buffer, and incubated for 15 min at 25°C in the dark in a reagent mix comprising 5 μl of the annexin-V-FITC conjugate and 10 μl of PI. The samples were analyzed using a flow cytometer (Novocyte, Agilent, United States) to determine the cell percentage at various phases.

For detecting lymphocyte subsets, 100 μl of the blood sample was stained with 10 μl of FITC-conjugated CD3, PE-conjugated CD4, and APC-conjugated CD8 reagent (Beckman Coulter), followed by incubation for 15 min in the dark for lysis of red blood cells. After washing, the cells were resuspended and subjected to flow cytometric analysis (Beckman). Lymphocytes were defined with their forward and side scatter characteristics. T lymphocytes were identified (CD3^+^) and then subdivided into CD4^+^ or CD8^+^ populations.

### Biochemistry analysis and ELISA

The ALT and AST levels in the culture medium supernatant or rats’ serum were determined using the ALT and AST assay kits (Nanjing Jiancheng Bioengineering Institute, China). The IL-6 and VCAM levels in the culture medium supernatant or rats’ serum were evaluated using rat IL-6 and VCAM-1 ELISA kits (Lunchangshuo Bio, China), respectively. Serum TPRO, ALB, TBIL, PCT, IL-10, ICAM-1, and CRP levels were examined using corresponding rat ELISA kits (Lunchangshuo Bio).

### qRT-PCR

The TRIzol reagent (Invitrogen, Carlsbad, United States) was used to isolate total RNA from cells and liver tissues. cDNA was synthesized using total RNA (2 μg), and Green Supermix (Roche, Basel, Switzerland) was used to conduct the real-time polymerase chain reaction (RT-PCR) on an ABI7900HT fast real-time PCR system (Applied Biosystems, CA, United States). GAPDH and β-actin was used as an internal control. The primer sequences used in qRT-PCR are listed in Table 2.

**TABLE 2:** Primer sequence for the study.

Gene	Primer	Sequence (5′-3′)
ALT	Forward	TGC​GGG​TTT​CGT​GGT​GGC​TAT
	Reverse	TCG​GAG​GGT​GTT​GGC​GGA​CT
AST	Forward	CGG​GAC​TTG​GTC​TCA​CAT​CAC​T
	Reverse	GGA​GGT​AGC​CAC​GTA​ATC​TAG​GTT​C
IL-6	Forward	TTC​ACA​GAG​GAT​ACC​ACC​CAC​AA
	Reverse	ACC​AGA​GCA​GAT​TTT​CAA​TAG​GCA
VCAM-1	Forward	CGG​CAT​TTA​TGT​ATG​TGA​AGG​GAT
	Reverse	TCT​TTG​ACG​CTC​TTA​GAT​GGG​AAG
ICAM-1	Forward	TCT​GTC​AAA​CGG​GAG​ATG​AAT​GG
	Reverse	TCT​GGC​GGT​AAT​AGG​TGT​AAA​TGG
CRP	Forward	CAT​CTG​TGC​CAC​CTG​GGA​GTC​T
	Reverse	AAA​GCC​ACC​GCC​ATA​CGA​GTC
IL-10	Forward	TGG​ACA​ACA​TAC​TGC​TGA​CAG​ATT​C
	Reverse	TCC​ACT​GCC​TTG​CTT​TTA​TTC​TC
GAPDH	Forward	GCC​TTC​CGT​GTT​CCT​ACC​CC
	Reverse	CGC​CTG​CTT​CAC​CAC​CTT​CT
β-actin	Forward	CCG​TAA​AGA​CCT​CTA​TGC​CAA​CA
	Reverse	GAG​CCA​CCA​ATC​CAC​ACA​GAG​T

### Immunoblotting

The target cells were used to isolate the proteins. The isolated proteins were deposited onto the PVDF membrane after electrophoresis with SDS-PAGE and then determined by immunoblotting with the specified primary antibodies. TLR4 (1/1000, ab13556, Abcam), NF-κB p65 (1/1000, 8242S, Cell Signaling Technology, Danvers, United States), p-NF-κB p65 (1/500, ab194726, Abcam), and actin (use concentration of 1 μg/ml, ab8226, Abcam, endogenous control) antibodies were used in this study. Antirabbit or antimouse IgG antibodies conjugated to horseradish peroxidase (Abcam) were used to cultivate the membranes. The protein bands were seen using the Thermo Scientific Pierce ECL Western Blotting Substrate.

### Animals and experiment design

Male SD rats (weighing 130–150 g, *n* = 24) were purchased from SLAC laboratory animal company (Changsha, China). All rats were kept under specific-pathogen-free (SPF) conditions with free access to food and drinking water at a room temperature of 22°C–25°C and humidity of (50.0 ± 2.0) %. The lights were turned on every 12 h for illumination. All procedures were approved by the animal ethics committee of Hunan University of Chinese Medicine (ZYFY20211111).

The rats were randomly allocated into four groups: control (*n* = 6), ACLF model (*n* = 6), ACLF model + AT-1 (*n* = 6), and ACLF model + AT-1 + PDTC (*n* = 6). The rats, except for the control group, were subcutaneously injected 0.5 ml of bovine serum mixed Freund’s incomplete adjuvant (contained 8 mg/ml bovine serum) on days 0, 14, 24, and 34. Then, the rats received tail vein injections 12 times (twice a week for 6 weeks) with bovine serum saline solution (5 mg/ml for 1^st^ injection, 6.25 mg/ml for 2^nd^ injection, 7.5 mg/ml for 3^rd^ injection, 8.75 mg/ml for 4^th^ injection, 10 mg/ml for 5th–12th injection, 400 μl) to generate an immune liver fibrosis model. For inducing the ACLF model, rats with immune liver fibrosis were fasted for 12 h and then intraperitoneally injected with LPS at a dose of 100 μg/kg and D-galactosamine (D-GalN) (Sigma-Aldrich, United States, G0500) at a dose of 400 mg/kg (Li et al., 2017). For PDTC treatment, PDTC (15 mg/kg) was intraperitoneally injected 30 minutes before the ACLF induction. For AT-1 treatment, AT-1 (20 mg/kg) was intraperitoneally injected 30 minutes before the ACLF induction.

Then, 12 h after D-GalN and LPS challenge, the rats were sacrificed under anesthesia. The blood samples were collected from the abdominal aorta, and the liver was also collected. The right leaf was fixed in 10% neutral buffered formalin, and the rest of the liver was cut into small pieces and frozen in liquid nitrogen for further study.

### Histological analysis

The liver specimens were embedded in paraffin and sectioned into 4-μm thick tissue slices. All sections were first stained with hematoxylin and eosin (H&E) for morphological structure analysis. For immunohistochemical staining (IHC), after antigen retrieval and endogenous peroxidase blocking, the sections were incubated with primary antibodies against TLR4 (1/100, 19811-1-AP, Proteintech, Wuhan, China), p-NF-κB p65 (1/50, ab194726, Abcam), IL-6 (use concentration of 1 μg/ml, ab9324, Abcam), and VCAM-1 (1/500, ab134047, Abcam) at 4 °C overnight and then incubated with secondary antibodies at room temperature for 2 h. Freshly prepared 3,3′-diaminobenzidine solution (Boster) was used for coloration. Next, the sections were counterstained with hematoxylin. The brown color indicated positive staining.

### Statistical analysis

The cell experiments were performed at least three times, and the animal experiments were performed at least six times. Experimental data from individual experiments were presented as the mean ± standard deviation (SD). Using GraphPad Prism software, comparisons between conditions were conducted by Student’s t-test or one-way ANOVA with Tukey’s test *post hoc* analysis. *p* < 0.05 was deemed as a statistically significant difference.

## Results

### Drug targets of the Wenyang Jiedu Huayu formula and disease targets of ACLF

**FIGURE 1:**
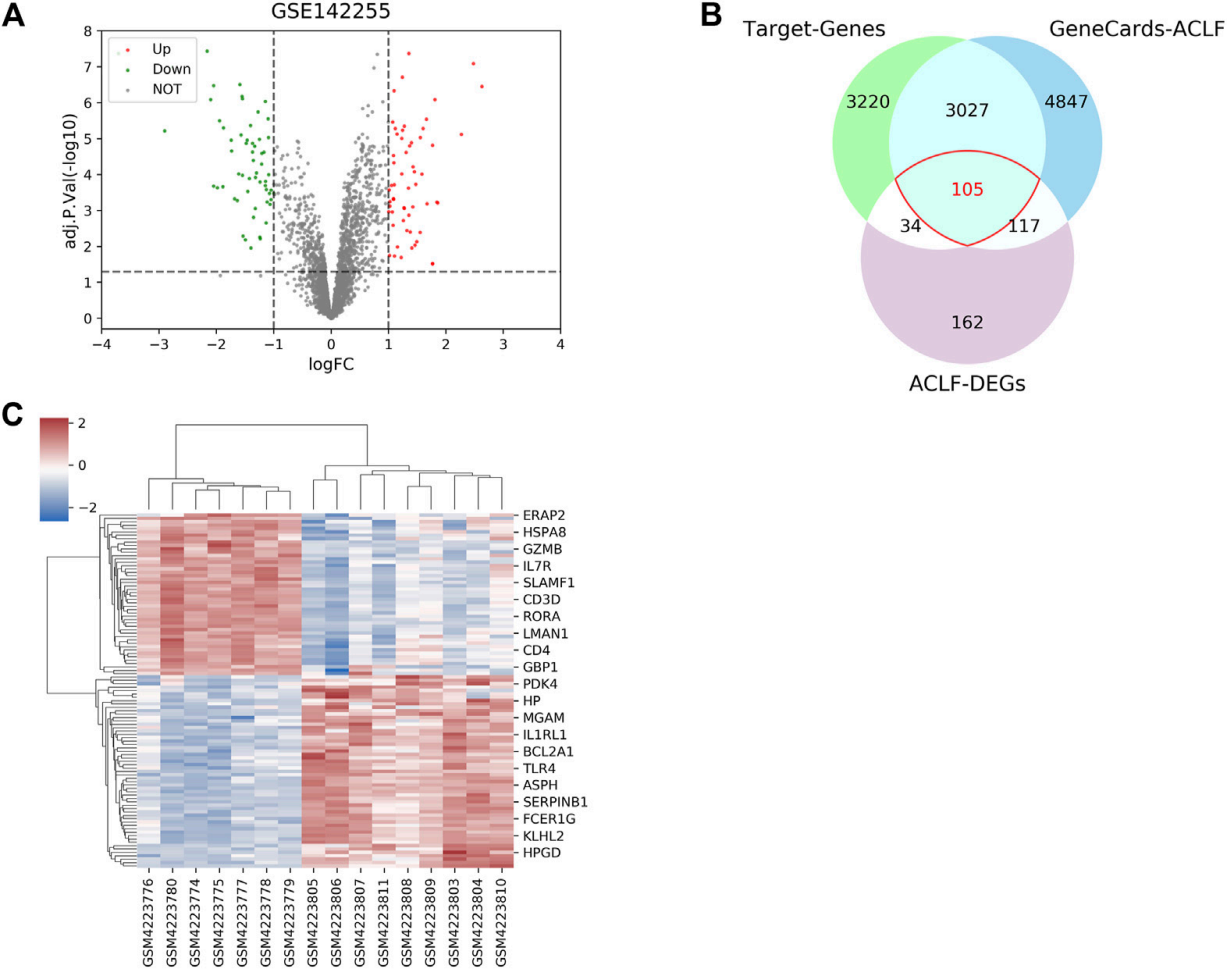
Drug targets of the Wenyang Jiedu Huayu formula and disease targets of acute-on-chronic liver failure **(ACLF). (A)** Differentially expressed genes (DEGs) between normal and ACLF samples according to GSE142255. **(B–C)** DEGs, drug targets retrieved from PubMed and PubChem, and disease targets retrieved from GeneCards were compared, and overlapping genes were further analyzed.

First, the active compounds of six main herbs of WYJDHY, namely, BaiZhu, Chishao, DanShan, YiYiRen, YinChen, and FuZi, were retrieved from TCMSP setting the threshold as OB ≥ 20% and DL ≥ 0.1. Those compounds were applied for PubChem analysis, and 23 compounds with targeted genes were obtained (Table 1). A total of 6,386 drug target genes were obtained based on 23 compounds. Regarding disease targets, 8,096 genes were associated with ACLF, according to the GeneCards database, among which 3,132 were drug targets obtained from the last step. Next, differentially expressed genes (DEGs) between normal and ACLF samples were analyzed according to GSE142255 (Figure 1A); using the thresholds of |logFC|>1 and adj.P.Val<0.05, a total of 105 DEGs were identified as the co-targets of drug and disease (Figure 1B–C, due to limited space, Figure 1C only showed the part of genes’ name; 105 DEGs are listed in Supplementary Table S1).

### Gene Ontology functional and KEGG signaling pathway enrichment annotation on 105 hub genes

**FIGURE 2:**
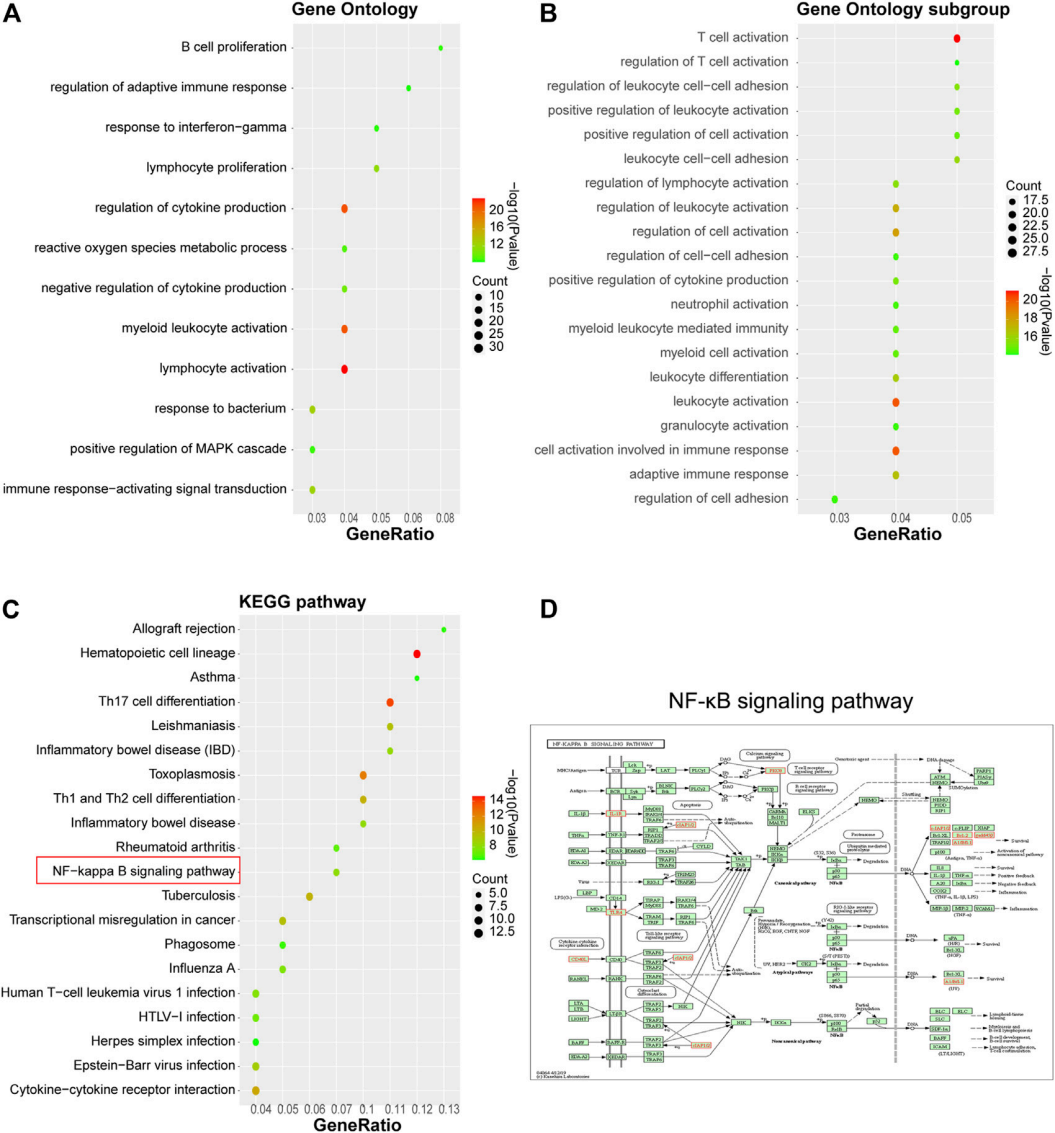
Gene Ontology functional and KEGG signaling pathway enrichment annotation on 105 hub genes. **(A)** GO analysis; **(B)** GO subgroup analysis; **(C)** KEGG analysis; and **(D)** NF-κB signaling pathway.

Functional and signaling pathway enrichment annotation was conducted upon these 105 DEGs using Metascape. As inferred from Figure 2, 105 DEGs were significantly associated with biological activities such as lymphocyte activation, reactive oxygen species metabolism, MAPK cascade regulation, and immune response regulation (Figures 2A,B) and pathways such as Th17 cell differentiation, cancer transcriptional dysregulation, phagosome, IBD, and NF-κB signaling (Figures 2C,D). Our previous study has confirmed that WYJDHY could modulate TLR4/NF-κB signaling during hepatic failure (Chen et al., 2013). Therefore, in the following investigation, we mainly focus on the active compounds that target NF-κB signaling.

### The herb–compound–signaling and herb–signaling–target networks

**FIGURE 3:**
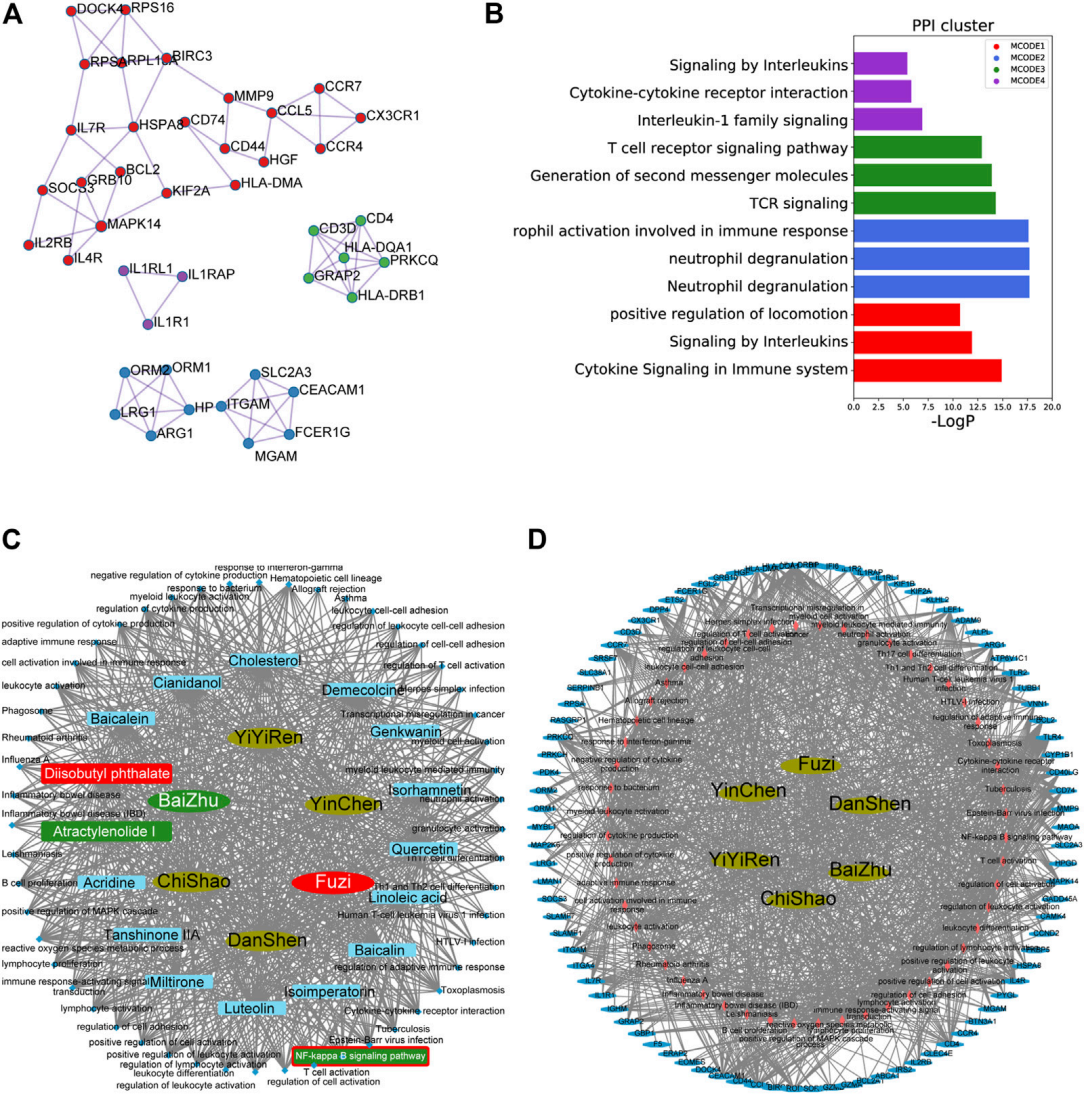
Herb–compound–signaling and herb–signaling–target networks. **(A–B)** Protein–protein interaction (PPI) analysis performed on 105 hub genes using STRING (https://string-db.org/cgi/input.pl) with physical score >0.132. **(C–D)** Herb–compound–signaling **(C)** and herb–signaling–target networks **(D)** were constructed by Cytoscape_v3.8.0.

Protein–protein interaction (PPI) analysis was performed on 105 hub genes using STRING (https://string-db.org/cgi/input.pl) with a physical score >0.132; Figures 3A,B show that 105 DEGs could be clustered into four functional modules. Cytoscape_v3.8.0 was used to construct the herb–compound–signaling–target networks (Smoot et al., 2011). The herbs, compounds, proteins, or pathways were represented by nodes in these graphical networks, whereas the interactions between the herb, compound, target, or signaling were represented by edges (Figure 3C–D, Supplementary Table S2, S3). A total of 16 active compounds could target 52 pathways. As inferred from Figures 3C,D and Supplementary Table S2, S3, the compounds AT-1 and diisobutyl phthalate, respectively, from warming Yang function herbs BaiZhu and FuZi, have been regarded as the hub compounds for targeting the NF-κB pathway. Among them, AT-1 has been reported to ameliorate acute liver injury in mice (Du et al., 2022). Therefore, we chose AT-1 for further investigation.

### Effects of AT-1 on LPS-induced toxicity in rat hepatocytes

**FIGURE 4:**
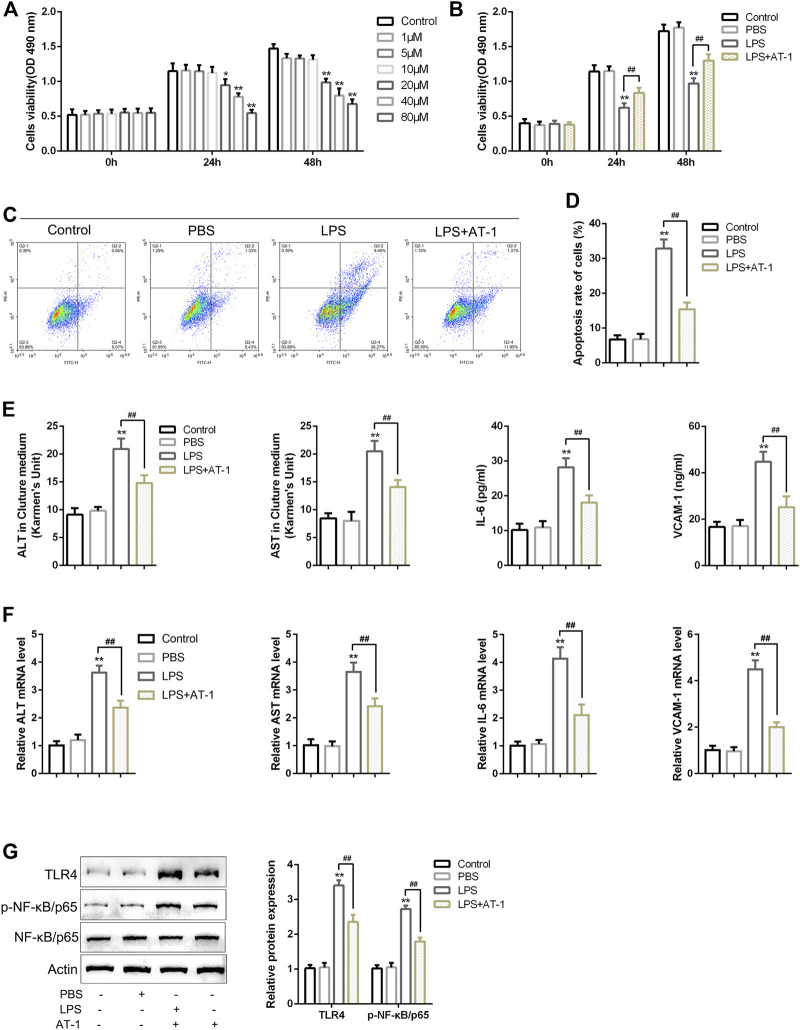
Effects of atractylenolide I (AT-1) on LPS-induced toxicity in rat hepatocytes. **(A)** BRL 3A cells were treated with gradient doses of AT-1 (0, 1, 5, 10, 20, 40, and 80 µM) for different time points and examined for cell viability using CCK-8 assays. Then, BRL 3A cells were treated with LPS in the presence or absence of 10 µM AT-1 and examined for cell viability using the CCK-8 assay **(B)**, cell apoptosis by flow cytometry **(C–D)**, the levels of ALT, AST, IL-6, and VCAM-1 in culture medium or cells using corresponding kits or qRT-PCR assay, respectively **(E–F)**, and the protein levels of TLR4, p-NF-κB/p65, and NF-κB/p65 by immunoblotting **(G)**. N = 3; *p < 0.05, **p < 0.01 compared to the control group; ##*p* < 0.01 compared to the LPS group.

For investigating the functions of AT-1, an LPS-induced toxicity model was placed in rat hepatocytes, the BRL 3A cell line. The target cells were treated for 24 and 48 h with gradient doses of AT-1 (0, 1, 5, 10, 20, 40, or 80 µM) and examined for cell viability to determine the minimum efficient concentration; as inferred from Figure 4A, 10 µM AT-1 did not affect the cell viability that was used in the following experiments. Then, BRL 3A cells were treated with LPS with or without 10 µM AT-1 and examined for 1 μg/ml LPS-stimulated toxicity. Figures 4B–D show that LPS stimulation inhibited cell viability and promoted cell apoptosis, which could be partially eliminated by AT-1 treatment. As for liver function markers and cytokines, LPS stimulation significantly increased the ALT, AST, IL-6, and VCAM-1 levels within the culture medium or cells, whereas AT-1 treatment partially reduced the contents of these factors (Figures 4E,F). Given the crucial role of NF-κB signaling in ACLF (Yang et al., 2014; Zhang et al., 2015; Zhou et al., 2018), the alterations in related factors were also investigated. As shown in Figure 4G, TLR4 protein levels were increased, and p65 phosphorylation was significantly promoted by LPS stimulation, whereas AT-1 treatment partially attenuated LPS effects on NF-κB signaling.

### NF-κB signaling is involved in AT-1 effects on LPS-induced toxicity in rat hepatocytes

**FIGURE 5:**
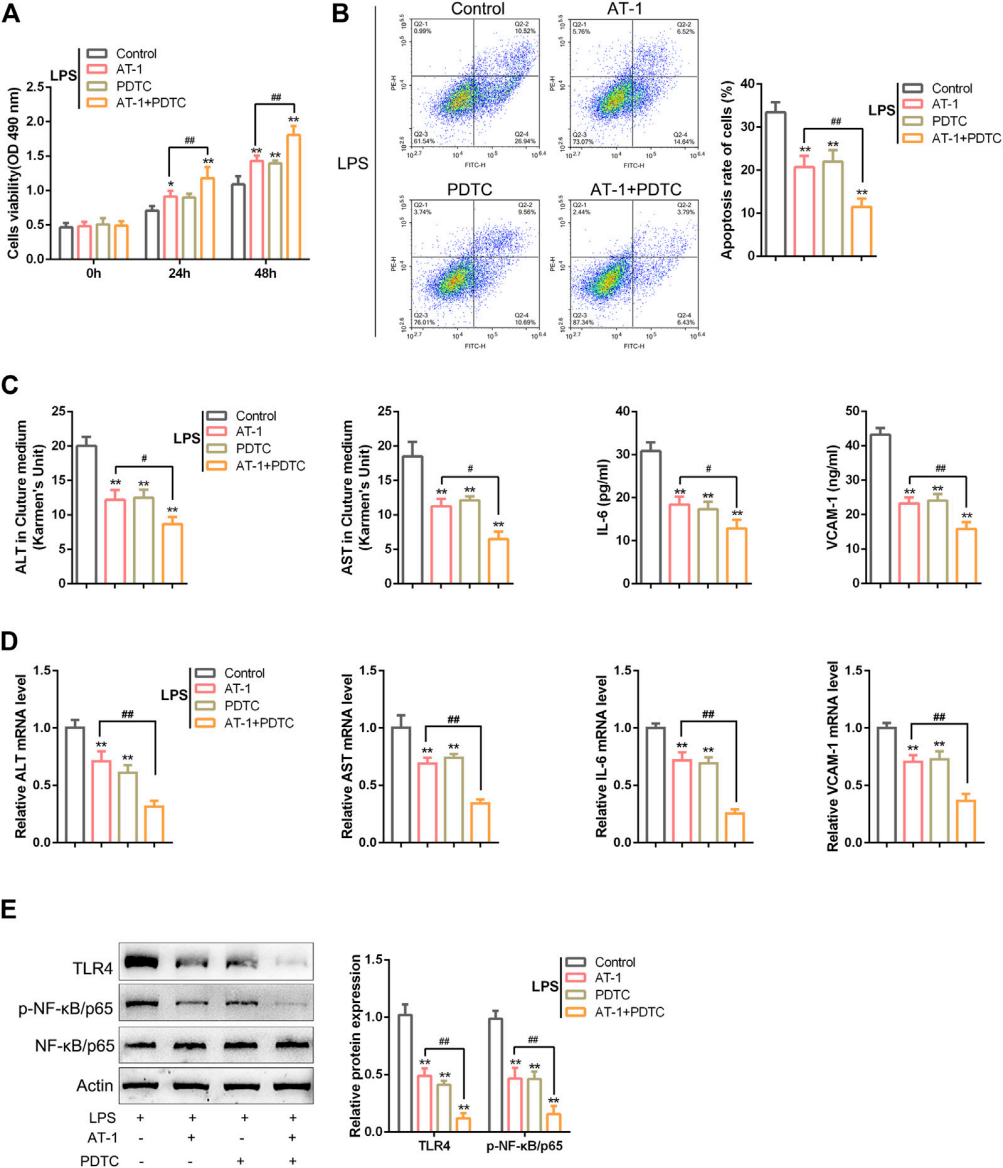
NF-κB signaling is involved in AT-1 effects on LPS-induced toxicity in BRL 3A cells. Target cells were treated with AT-1 (10 µM) and/or PDTC (the NF-κB signaling inhibitor, 10 µM) under LPS stimulation and examined for cell viability using CCK-8 assays **(A)**, cell apoptosis by flow cytometry **(B)**, levels of ALT, AST, IL-6, and VCAM-1 in the culture medium or cells using corresponding kits or qRT-PCR assay, respectively **(C–D)**, and protein levels of TLR4, p-NF-κB/p65, and NF-κB/p65 by immunoblotting **(E)**. N = 3; *p < 0.05, and **p < 0.01 compared to the control group; #*p* < 0.05 and ##*p* < 0.01 compared to the AT-1 group.

Regarding the involvement of NF-κB signaling, the dynamic effects of AT-1 and/or the NF-κB signaling inhibitor PDTC on BRL 3A cells were investigated. The target cells were treated with AT-1 (10 µM) and/or PDTC (10 µM) under LPS stimulation and examined for LPS-induced toxicity. Under LPS stimulation, single AT-1 or PDTC treatment dramatically enhanced cell viability but suppressed cell apoptosis, and the alterations were further amplified by combining AT-1 and PDTC (Figures 5A,B). Under LPS stimulation, single AT-1 or PDTC treatment remarkably decreased the ALT, AST, IL-6, and VCAM-1 levels within the culture medium or cells, whereas the combination of AT-1 and PDTC reduced the ALT, AST, IL-6, and VCAM-1 levels even more (Figures 5C,D). Regarding the alterations in NF-κB signaling, single AT-1 or PDTC treatment decreased TLR4 proteins and inhibited p65 phosphorylation, whereas AT-1 and PDTC combination exerted more inhibitory effects on NF-κB signaling (Figure 5E).

### NF-κB signaling is involved in AT-1 protection on the ACLF rat model

**FIGURE 6:**
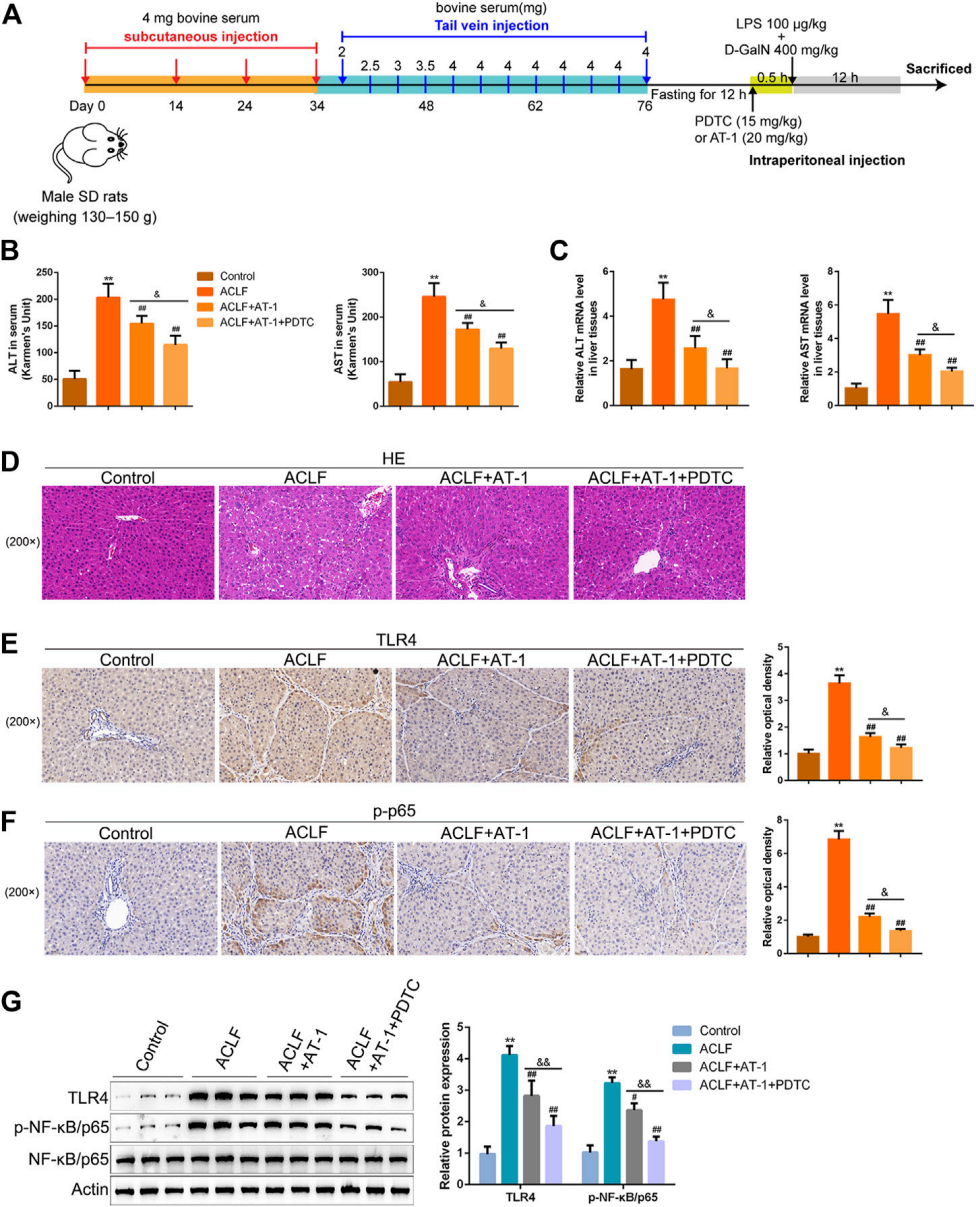
NF-κB signaling is involved in AT-1 protection in the acute-on-chronic liver failure (ACLF) rat model. **(A)** Schematic diagram showing the procedures of the ACLF rat model and AT-1 or PDTC treatment. The rats were randomly allocated into control (*n* = 6), ACLF model (*n* = 6), ACLF model + AT-1 (n = 6), and ACLF model + AT-1 + PDTC (*n* = 6) groups and treated accordingly. **(B–C)** Levels of ALT and AST were determined using ELISA in the serum or qRT-PCR assay in liver tissues. **(D)** Histopathological changes in liver tissues were evaluated by H&E staining. **(E–F)** Levels of TLR4 and p-p65 in liver tissues were evaluated by immunohistochemical staining (IHC). **(G)** Levels of TLR4, p-p65, and p65 in liver tissues were evaluated by immunoblotting. N = 6; **p < 0.01 compared to the control group; ##*p* < 0.01 compared to the ACLF model group; &*p* < 0.05 and &&*p* < 0.01 compared to ACLF+AT-1 group.

For validating the effects and mechanism of AT-1 on ACLF *in vivo*, a rat model was established as described, and treatments were administrated accordingly (Figure 6A). However, 12 h after the acute attacks, the ALT and AST levels in the serum or liver tissues were significantly elevated in ACLF model rats, whereas lowered by single AT-1 treatment or the combination of AT-1 and PDTC treatments compared with the model rats; furthermore, the combination significantly decreased the ALT and AST levels compared with single AT-1 treatment (Figures 6B,C). Liver function evaluation was further evidenced by histopathological examinations. In ACLF model rats, H&E staining shows that hepatic fibrosis was evident, pseudolobules were widely developed, hepatocytes were disordered, and necrosis was severe; the following symptoms were also observed: a large number of inflammatory cell infiltration, hepatic sinusoidal dilatation, and bleeding, with certain liver tissues displaying major or submassive necrosis (Figure 6D). These pathological changes were ameliorated by single AT-1 treatment or the combination of AT-1 and PDTC treatments (Figure 6D).

**FIGURE 7:**
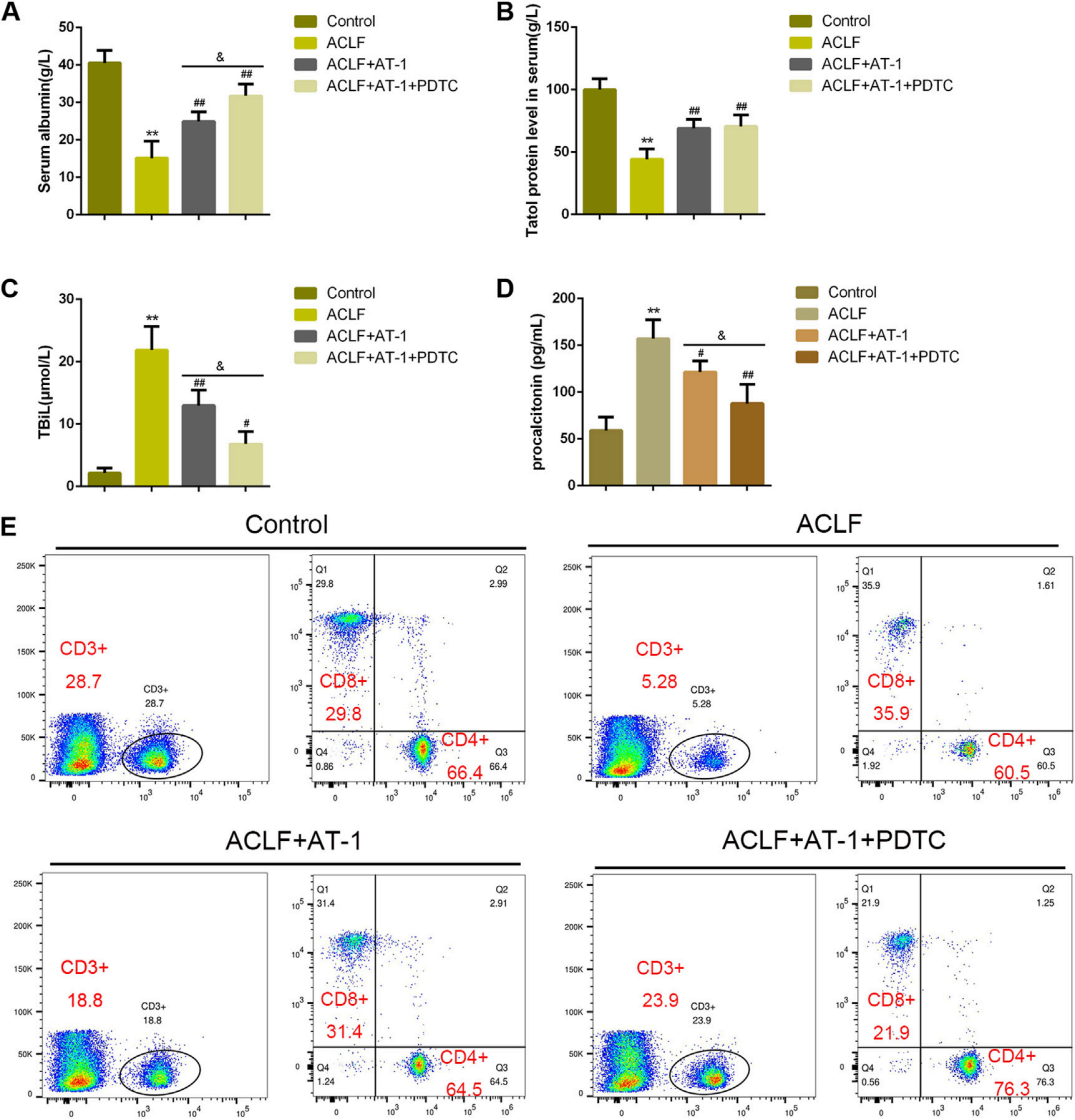
NF-κB signaling is involved in AT-1 effects on inflammation in model rats. **(A–D)** Serum ALB, TPRO, TBIL, and PCT were examined using ELISA. **(E)** Lymphocyte subsets were identified by flow cytometry. N = 6; **p < 0.01 compared to the control group; #*p* < 0.05, ##*p* < 0.01 compared to the ACLF model group; &&*p* < 0.01 compared to ACLF+AT-1 group.

**FIGURE 8:**
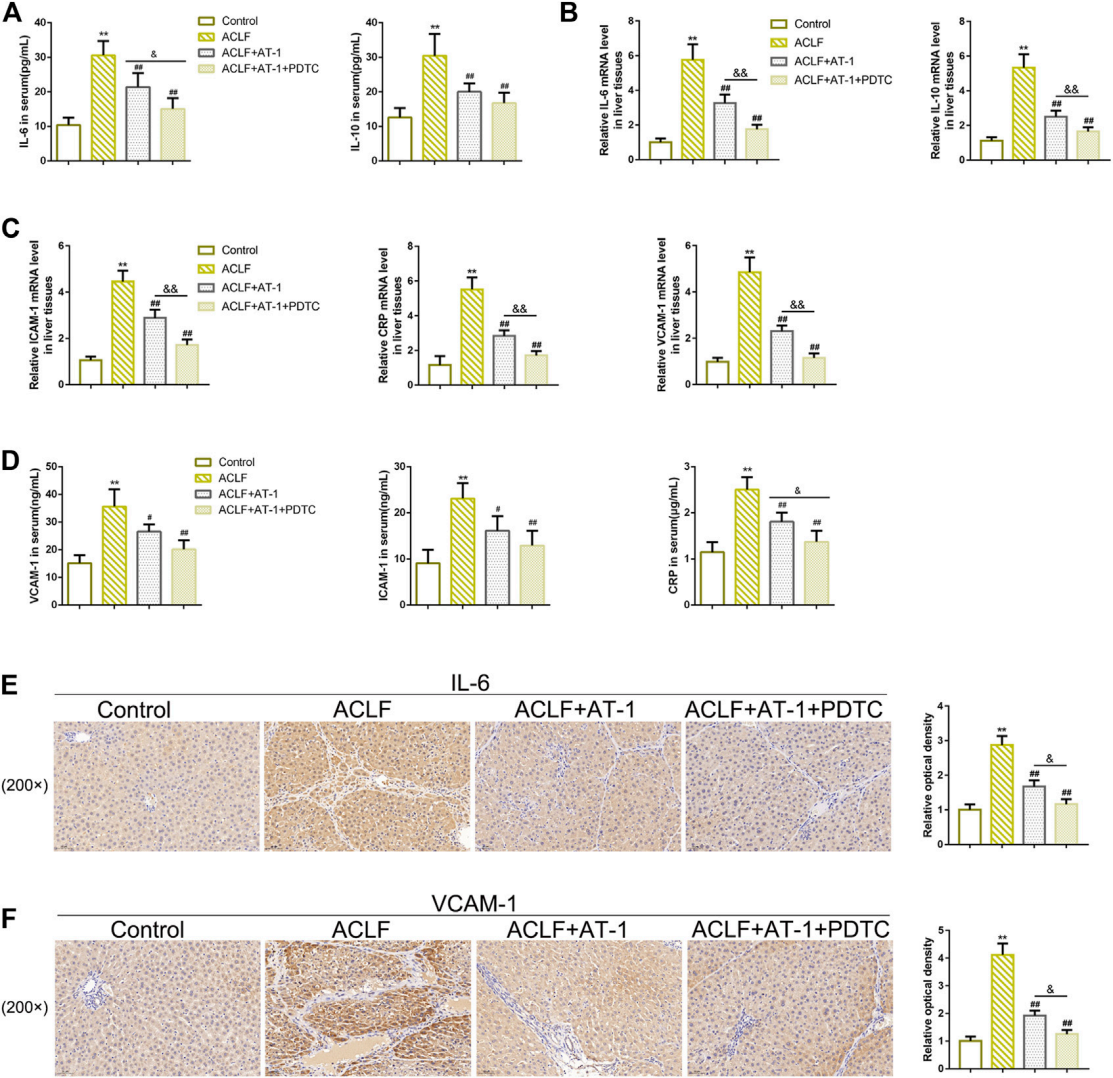
NF-κB signaling is involved in AT-1 effects on inflammation in model rats. **(A–D)** Levels of IL-6, IL-10, VCAM-1, ICAM-1, and CRP were examined using ELISA in the serum or qRT-PCR assay in liver tissues. **(E–F)** Levels of IL-6 and VCAM-1 in liver tissues were examined by IHC staining. N = 6; **p < 0.01 compared to the control group; ##*p* < 0.01 compared to the ACLF model group; &*p* < 0.05 and &&*p* < 0.01 compared to ACLF+AT-1 group.

Regarding local inflammation, the levels of TLR4 and p-p65 were dramatically increased in liver tissues of ACLF model rats, whereas partially decreased by single AT-1 treatment or the combination of AT-1 and PDTC treatments; moreover, the combination significantly decreased the TLR4 and p-p65 levels compared with single AT-1 treatment (Figures 6F,G). Furthermore, the serum of rats was collected for ELISA. ALB and TPRO were decreased, but TBIL and PCT were increased in ACLF rats, whereas opposite trends of these indexes were observed in single AT-1 treatment or the combination of AT-1 and PDTC groups (Figures 7A–D). Similarly, the combination of AT-1 and PDTC further amplified the improving effects of single AT-1 treatment on these indexes (Figures 7A–D). Next, lymphocyte subgroups were examined. Total CD3^+^ T-cell counts were considerably lower in ACLF rats, with the increase in the CD8^+^ subset and decrease in the CD4^+^ subset; single AT-1 treatment or the combination of AT-1 and PDTC increased CD3^+^ T-cell counts, with the decrease in the CD8^+^ subset and increase in the CD4^+^ subset (Figure 7E). Last, the levels of IL-6, IL-10, VCAM-1, ICAM-1, and CRP in the serum or liver tissues were examined, and the levels of IL-6 and VCAM-1 in liver tissues were detected. Consistent with local inflammatory changes, serum and tissue IL-6, IL-10, VCAM-1, ICAM-1, and CRP and tissue IL-6 and VCAM-1 levels were significantly increased in ACLF rats; single AT-1 treatment or the combination of AT-1 and PDTC remarkably decreased the levels of these inflammatory factors, and the combination of AT-1 and PDTC decreases these factors more (Figures 8A–F).

## Discussion

In this study, 23 compounds with 6,386 drug targets were obtained, and 8,096 genes were identified as ACLF disease targets, according to the GeneCards database, among which 3,132 were overlapping co-targets. Expression profile analysis identified 105 DEGs among the co-targets. These 105 DEGs were significantly associated with biological activities such as lymphocyte activation, reactive oxygen species metabolism, MAPK cascade regulation, immune response regulation, and pathways such as Th17 cell differentiation, cancer transcriptional dysregulation, phagosome, IBD, and NF-κB signaling. After PPI analysis and network construction, AT-1 from the warming Yang herb BaiZhu has been identified as the hub active ingredient of the WYJDHY formula that could target and regulate NF-κB signaling. In cellular experiments, LPS stimulation inhibited rat hepatocyte viability, promoted cell apoptosis, increased the ALT, AST, IL-6, and VCAM-1 levels within the culture medium, and activated NF-κB signaling, whereas AT-1 treatment significantly attenuated LPS-induced toxicity on BRL 3A cells. Furthermore, the NF-κB signaling inhibitor PDTC exerted similar effects on LPS-stimulated BRL 3A cells to those of AT-1, and the combination of PDTC and AT-1 further attenuated LPS-induced toxicity on BRL 3A cells. *In vivo*, single AT-1 or the combination of PDTC and AT-1 significantly improved liver dysfunctions and local inflammation in ACLF model rats; the combination of PDTC and AT-1 improved the symptoms more.

The protective functions of the six main herbs of WYJDHY on liver disorders have been reported previously. *Paeonia lactiflora* Pall. (PLP), also named Chishao, exerted an anti-inflammatory effect on the alpha-naphthylisothiocyanate (ANIT)-induced cholestasis model (Ma et al., 2018). FuZi, the monarch drug of another TCM formula Fuzi Lizhong decoction, exerted protective effects against non-alcoholic fatty liver disease (NAFLD) through anti-inflammatory responses (Yang et al., 2018; Yang et al., 2020). Single Yinchen or the combination of Yinchen with other herbs could protect against hepatocyte apoptosis (Zhang et al., 2022a), non-alcoholic fatty liver disease (Guo et al., 2017), or ANIT-induced cholestatic liver injury (Su et al., 2021). Compounds such as salvianolic acid C (Wu et al., 2019) and salvianolic acid A protect against drug-induced acute liver failure or CCl_4_-induced liver fibrosis. Pan et al. (2021) demonstrated the liver-protective effects of Shenling Baizhu San against inflammatory damage through TLR4/NLRP3 signaling in rats with NAFLD. In this study, 23 main compounds from six herbs were obtained; these herbs might target 3,132 of the 8,096 ACLF disease targets *via* expression profile analysis. Of the 3,132 co-targets, 105 have been found to be differentially expressed between ACLF and normal samples.

For identifying the main functions and signaling pathways that these compounds might affect, these 105 DEGs were applied for GO functional and KEGG signaling pathway enrichment annotation. GO annotation showed that they were significantly associated with biological activities such as lymphocyte activation, reactive oxygen species metabolism, MAPK cascade regulation, and immune response regulation, whereas KEGG pathway annotation showed that they were remarkably enriched in Th17 cell differentiation, cancer transcriptional dysregulation, phagosome, IBD, and NF-κB signaling. As aforementioned, the NF-κB pathway, a vital component of TLRs, exerts a crucial effect on regulating ACLF severity and prognosis (Hayden and Ghosh, 2008; Sun et al., 2009; McCabe et al., 2012; Yang et al., 2014; Zhang et al., 2022b). Our previous study has confirmed that WYJDHY could modulate TLR4/NF-κB signaling during hepatic failure [23]. Several reagents have been reported to alleviate ACLF through regulating the NF-κB pathway, including trichostatin A (Zhang et al., 2015), ginsenoside Rg1 (Zhao et al., 2021), and plumbagin (Wang H. et al., 2016). Considering that the six main herbs exert their protective effects against liver disorders through regulating inflammatory signaling or factors, GO and KEGG annotation results were consistent with the etiology of ACLF, suggesting that the 23 major active compounds might exert their functions *via* inflammatory signaling, particularly the NF-κB pathway. After PPI analysis and network construction, AT-1 from the warming Yang herb BaiZhu had been identified as the hub active ingredient of the WYJDHY formula for targeting the NF-ΚB pathway and was chosen for following cellular experiments.

Reportedly, in an acetaminophen-induced acute liver failure mouse model, both LPS-TLR4 pathway therapies reduced cell death, increased renal function, and decreased the coma-free survival rate (Shah et al., 2013), suggesting that the LPS-TLR4 axis is implicated in inflammatory processes that lead to tissue damage, and blocking this signaling pathway stops disease development. Therefore, in this study, an LPS-induced toxicity model was established in rat hepatocyte BRL 3A cells, and the effects of AT-1 were investigated. Consistent with previous studies, LPS stimulation inhibited cell viability, enhanced cell apoptosis, and promoted the levels of ALT, AST (liver function markers), IL-6, and VCAM-1 (cytokines) (Shah et al., 2013; Chen et al., 2018; Wu et al., 2021; Zhang et al., 2021). As expected, AT-1 treatment significantly ameliorated LPS-induced toxicity on BRL cells by rescuing cell viability, suppressing cell apoptosis, and decreasing ALT, AST, IL-6, and VCAM-1 levels. Considering the critical roles of the NF-κB pathway, a vital part of TLRs, in ACLF (Hayden and Ghosh, 2008; Yang et al., 2014; Zhang et al., 2022b), the effects of AT-1 on the NF-κB pathway were investigated in LPS-stimulated BRL 3A cells. Consistently, LPS-induced activation could be significantly inhibited by AT-1 treatment. Moreover, the combination of PDTC, the NF-κB pathway inhibitor, and AT-1 further attenuated LPS-induced toxicity on BRL 3A cells and NF-κB pathway activation, suggesting that AT-1 might exert the hepatic protective effects through inhibiting the NF-κB pathway.

The most common cause of ACLF is liver fibrosis/cirrhosis. Continuous inflammation, SIRS, and cytokine storms result from acute damage caused by alcohol use, hepatotropic viral infection, or medications; they play a key role in the pathophysiology of liver failure and subsequent organ failure (Sarin and Choudhury, 2016). Rat serum levels of ALT, AST, TBIL, and PCT increased dramatically after injection with LPS/D-GalN, and a considerable number of infiltrating inflammatory cells and necrotic hepatocytes were identified in liver tissues (Li et al., 2017; Sarin et al., 2019). These findings matched the clinical and pathological abnormalities seen in ACLF, showing that the ACLF rat model was built successfully. The liver functions improved after treatment with AT-1 alone or in conjunction with PDTC, as manifested by decreased ALT, AST, TBIL, and PCT levels and increased histopathological features of liver tissues. Furthermore, AT-1 alone or in conjunction with PDTC raised CD3^+^ T-cell numbers while decreasing CD8^+^ subsets and increasing CD4^+^ subsets. In the progression of ACLF, the TLR4 signaling pathway is stimulated, and the production of NF-κB is increased in the context of local inflammation. The NF-κB pathway has long been considered a prototypical pro-inflammatory signaling pathway, largely based on the role of NF-κB in the expression of pro-inflammatory genes, including cytokines, chemokines, and adhesion molecules (Lawrence, 2009). Pro-inflammatory factors such as IL-1, IL-18, IL-6, and TNF-α are released *via* the NF-κB-mediated inflammatory cascade (Moreau, 2016). Zhang et al. (2022b) elucidated that cylindromatosis regulates IL-6 by inhibiting NF-ĸB. Zhang et al. (2019) revealed that methane ameliorates cognitive dysfunction by promoting IL-10 expression and inhibiting the NF-κB pathway in aging mice. In this investigation, single AT-1 or in conjunction with PDTC reduced the levels of these inflammatory factors considerably, and with the combination of AT-1 and PDTC, lowering them more. Therefore, AT-1 might help ameliorate symptoms and local inflammation in ACLF model rats, and NF-κB signaling might play a role.

In conclusion, 23 active ingredients of six herbs in the WYJDHY formula were retrieved, and 105 co-targets of drugs and disease were identified to be differentially expressed between ACLF and normal tissues. AT-1 protects against LPS-induced toxicity on hepatocytes, and the NF-κB pathway might be involved.

## Data Availability

Publicly available datasets were analyzed in this study. These data can be found at: https://www.ncbi.nlm.nih.gov/geo/query/acc.cgi?acc = GSE142255.
