# Corrigendum: Network pharmacology-based prediction of active compounds in Wenyang Jiedu Huayu formula acting on acute-on-chronic liver failure with experimental support *in vitro* and *in vivo*

**DOI:** 10.3389/fphar.2022.1105804

**Published:** 2022-12-01

**Authors:** Dan Tang, Ruo-Yu Wang, Ke-Wei Sun, Yunan Wu, Lin Ding, Yang Mo

**Affiliations:** ^1^ Department of Hepatology, The First Hospital of Hunan University of Traditional Chinese Medicine, Changsha, China; ^2^ Academic Affairs Office, Hunan University of Traditional Chinese Medicine, Changsha, China

**Keywords:** acute-on-chronic liver failure, Wenyang Jiedu Huayu formula, network pharmacology, atractylenolide I, rat hepatocytes, ACLF rat model

In the published article, there was an error in the **Funding statement**. The funding statement for the National Natural Science Foundation of China was displayed as “(8197152432 and 82104809).” The correct Funding statement appears below.

“Funding

This study was supported by the National Natural Science Foundation of China (81973833 and 82104809), and the Hunan University of Chinese Medicine Graduate student innovation topics (2020CX18).”

The authors apologize for this error and state that this does not change the scientific conclusions of the article in any way. The original article has been updated.

